# Indole hybridized diazenyl derivatives: synthesis, antimicrobial activity, cytotoxicity evaluation and docking studies

**DOI:** 10.1186/s13065-019-0580-0

**Published:** 2019-05-10

**Authors:** Harmeet Kaur, Jasbir Singh, Balasubramanian Narasimhan

**Affiliations:** 1 Faculty of Pharmaceutical Sciences, Maharshi Dayanand University, Rohtak, 124001 India; 2 College of Pharmacy, Postgraduate Institute of Medical Sciences, Rohtak, 124001 India

**Keywords:** Diazenyl, Indole, Antimicrobial, Cytotoxicity, Docking

## Abstract

**Background:**

In search of effective antimicrobial and cytotoxic agents, a series of indole hybridized diazenyl derivatives (**DS-1** to **DS-21**) was efficiently prepared by condensation of diazotized *p*-aminoacetophenone with indole or nitroindole followed by reaction with different aromatic/heteroaromatic amines of biological significance. The synthesized derivatives were characterized by various spectroscopic techniques.

**Methodology:**

The antimicrobial evaluation of **DS-1** to **DS-23** was done by tube dilution method against various pathogenic bacterial and fungal strains. The active antimicrobial derivatives were further evaluated for cytotoxicity against human lung carcinoma cell line (HCT-116), breast cancer cell line (MDAMB231), leukemic cancer cell line (K562), and normal cell line (HEK293) by MTT assay using doxorubicin as the standard drug. The test derivatives were additionally docked for the B-subunit of enzyme DNA gyrase from *E. coli* at the ATPase binding site to study the molecular interactions using Schrodinger maestro *v11.5* software.

**Results and discussion:**

Most of the synthesized derivatives have shown high activity against Gram-negative bacteria particularly *E. coli* and *K. pneumonia* with MIC ranging from 1.95 to 7.81 μg/ml. The derivatives have demonstrated very less activity against tested Gram positive bacterial and fungal strains. The derivatives **DS-14** and **DS-20** have been found to active against breast cancer cell line and human colon carcinoma cell line having IC_50_ in the range of 19–65 µg/ml. All the derivatives were found to less potent against leukemic cancer cell line. The synthesized derivatives have revealed their safety by exhibiting very less cytotoxicity against the normal cell line (HEK-293) with IC_50_ > 100 µg/ml. Most of the active derivatives have shown good docking scores in comparison to the standard drugs against DNA gyrase from *E. coli*. Further ADME predictions by Qikprop module of the Schrodinger confirmed these molecules have drug like properties.

**Conclusion:**

The derivatives **DS-14** and **DS-20** have shown potential against Gram-negative bacteria and breast cancer cell line and can be used as a lead for rational drug designing of the antimicrobial and cytotoxic agents.
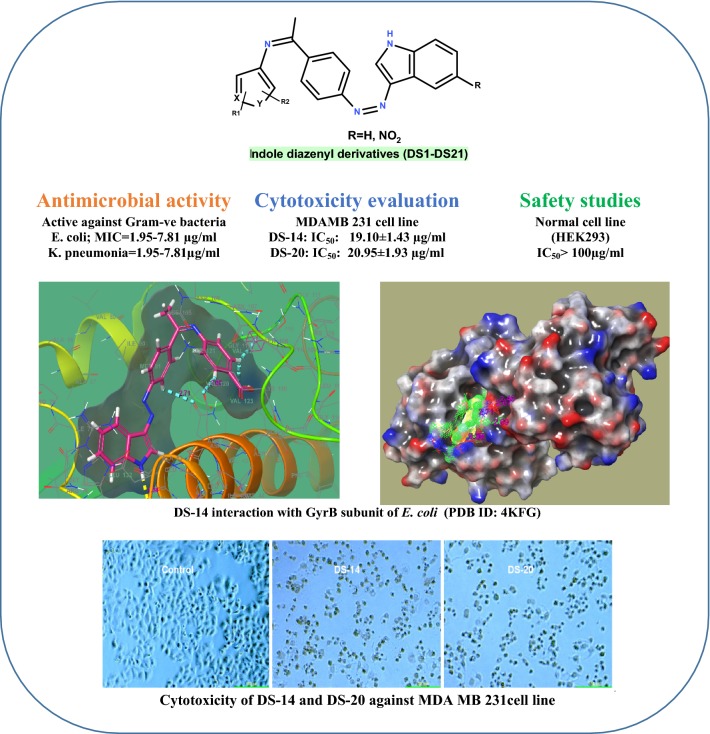
.

## Introduction

The burgeoning number of the infectious diseases due to the growing concerns of the antimicrobial resistance (AMR) has been presented the major threat to the existence of the mankind [1, 2]. The World Health Organization report in 2016 disclosed that tuberculosis, diarrhoea and respiratory infections are among the top ten diseases with the accountability of approximately 5.7 million deaths worldwide [3]. A recent report estimated that the microbial infections would cause 10 million deaths annually by 2050 [4]. The treatment options for the contagious diseases conferred a complicated mystery in the early 1900, but it was unlocked by the accidental discovery of the penicillin in 1928, the first antibiotic, by Alexander Fleming, resulted in the beginning of antibiotic era [5]. Afterwards, the modern medicine have been revolutionized by the antibiotics and blessed the millions of human lives. But the overproduction, inappropriate and extensive use, poor infection control, poor hygiene and sanitation, discovery of fewer new antibiotics have led to the development of antimicrobial resistance [6]. From the late 1960 to early 1990, various new antibiotics have been introduced by the pharmaceutical industry to conquer the drug resistance dilemma, but later on the number of investigating antibiotics in clinical trials dramatically decreased and only a bunch of new antibiotics have been introduced in the market. The concern of pharmaceutical industry has been also shifted from the discovery of antimicrobials to the drugs dealing with other lethal diseases due to socio-economic and financial factors [7–9]. According to a study by the Pew Charitable Trusts in 2016, each currently available antibiotic is derived from a pre-existing class discovered by 1984 [10]. Hence the post-antibiotic era will represent the future panorama of the world with the high mortality rates due to the common infections. This situation has been further inflamed as most of the other malignant diseases with the host immune-compromised or concomitant illness especially cancer often accompanied by the microbial infections [11, 12]. The cancer patients are at the higher risk of microbial infections as compared to the normal persons due to the easy access of the microorganisms as a result of interrupted epithelial barriers, compromised host defence, the absence of neutrophils, and shifts in the microbial flora, etc. [13, 14]. Mostly patients diagnosed with cancer are also recommended with the antibiotics to increase the life span. The most lethal cancers have also developed resistance to the current chemotherapeutic agents hence presented the limited treatment scope [15]. All above facts necessitates the need for the development of new antimicrobial and cytotoxic agents and also signifies the importance of understanding the mechanisms of drug interactions on a molecular level to further enhance the development of new antimicrobial and anticancer agents.

DNA gyrases are well-studied drug targets that are present in almost all bacteria and are essential for growth of bacteria [16, 17]. The low structural homology exhibited by these enzymes with human topoisomerases make them attractive drug targets for antibacterial therapy. Mostly these enzymes consist of two subunits. The B-subunit of DNA gyrase (GyrB) consist of ATP binding pockets which are responsible for the DNA replication. The small molecules inhibition of this pocket is plausible, and several lead compounds have been developed by targeting this pocket [18, 19].

**Fig. 1:**
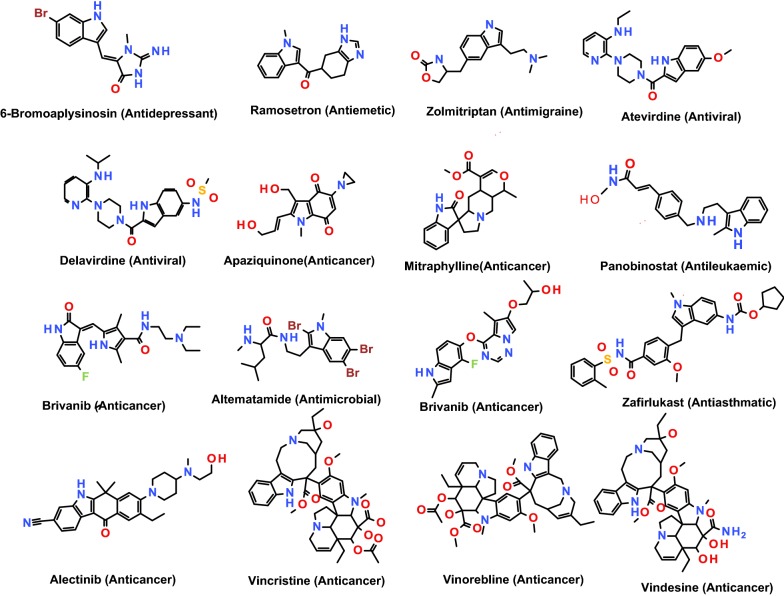
Indole containing drug molecules

The indole derivatives have been emerged as the drugs of immense importance in the recent times and well known for their significant biological activities such as cytotoxic, antimicrobial, antidiabetic, and anti-inflammatory activities [20–23]. Several indole containing drugs are available in the market. Some of the indole containing drugs have been listed in Fig. 1.

Likewise, diazenyl compounds characterized by the presence of azo linkage (–N=N–) have also attracted the attention of the researchers due to their significant biological activities. Many diazenyl derivatives (i.e. diazeniumdiolate prodrugs, diazenecarboxamides, diazenyl complexes etc.) have been accounted for their cytotoxic potential towards different cancerous cell lines in the recent years [24–26]. These derivatives also reported to have antimicrobial activity [27, 28].

In perspective of above details, in the current study we have planned to synthesize the novel hybridized indole diazenyl derivatives using different aromatic/heteroaromatic amines of biological significance and evaluate their antimicrobial potential against various pathogenic strains and also against a number of cancerous cell lines for evaluation of their cytotoxic potential. Additionally, these derivatives also have been planned to study for the molecular interactions with the B-subunit of enzyme DNA gyrase at the ATP binding pocket.

## Results and discussion

### Chemistry

**Fig. 2:**
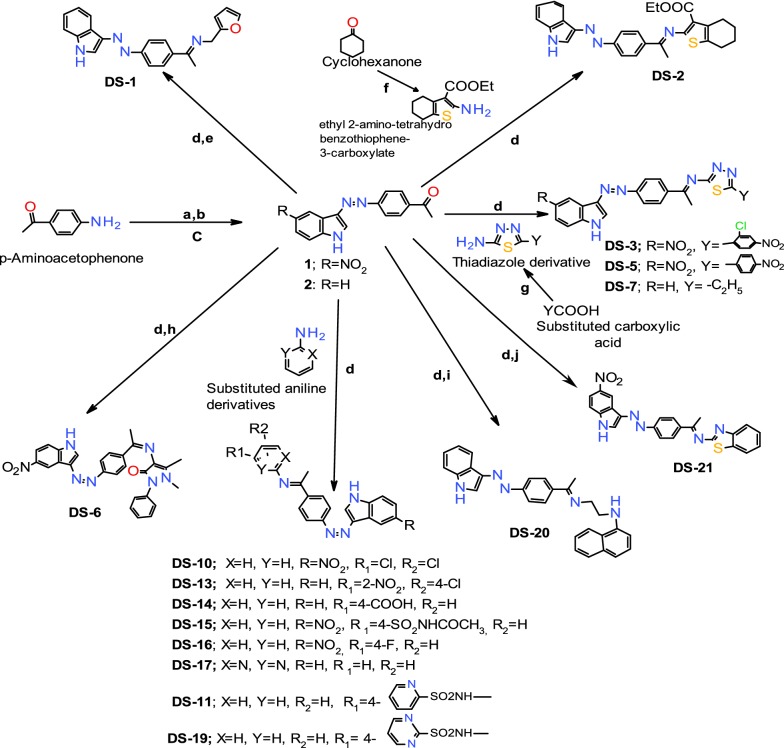
Synthetic methodology for Indole diazenyl derivatives (**DS1**–**DS-21**). Reagents and conditions: (a) NaNO_2_/HCl, 0 °C, (b) indole/nitroindole, acetic/propionic acid mixture (8:2), 0 °C, (c) Na_2_CO_3_, (d) ethanol, 5–7 drops of acetic acid, reflux for 7–8 h, (e) furfurylamine, (f) ethyl acetoacetate, triethylamine, sulphur, stirring for 10–15 h at RT, (g) POCl_3,_ thiosemicarbazide, reflux for 6–7 h at 70 °C, (h) 4-aminoantipyrine, (i) *N*-1-napthylethylenediamine, (j) 2-benzothiazole amine

The target compounds (**DS1**–**DS21**) were synthesized from the commercially available *p*-aminoacetophenone which was diazotized in the presence of NaNO_2_ and HCl followed by condensation with unsubstituted indole or nitroindole and further reaction with various amines of biological significance (Fig. 2). The amine, tetrahydrobenzothiophene used for synthesis of derivative **DS-2** was prepared by the Gewald reaction [29]. The amines used for the synthesis of **DS-3**, **DS-5** and **DS-7** were prepared by the reaction of aliphatic/aromatic carboxylic acids with the POCl_3_ and thiosemicarbazide as per the reported methods [30, 31]. The compounds **DS-4**, **DS-8**, **DS-9**, **DS-12** and **DS-18** were not mentioned in the scheme as these compounds do not meet the criteria of purity for the synthesized compounds. The structures of test compounds have been verified by IR, ^1^H-NMR, ^13^C-NMR, and mass spectroscopy.

The IR spectrum of synthesized compounds was determined by KBr pellet method. The NH stretch due to indole moiety was observed at 3217–3447 cm^−1^. The C=O group in dyes **1** and **2** was detected between 1668 and 1669 cm^−1^ respectively which was shifted to the 1601–1641 cm^−1^ in **DS-1** to **DS-23** indicating the formation of Schiff base (–CH=N– linkage). The aliphatic stretch was perceived in the range of 2847–3169 cm^−1^. The compound having ester group (**DS-2**) exhibited –C=O stretching at the 1721 cm^−1^. The –C=C– stretch of the aromatic rings appeared at 1512–1596 cm^−1^. The presence of band at 1401–1468 cm^−1^ confirmed the presence of azo linkage. The other peaks observed are the C–N stretching between 1011 and 1335 cm^−1^, Ar–O stretching at 1108–1278 cm^−1^, –C=C– bending at 682–747 cm^−1^, and C–S stretching at 617–701 cm^−1^. The NO_2_ stretch confirmed by the two strong bands at 1319–1338 cm^−1^ and 1468–1517 cm^−1^. The bands in the range of 535–1053 cm^−1^ have been assigned to the C-X (halogen) absorption. The proton NMR spectra of synthesized compounds were taken in DMSO at 400 MHz. The proton spectra of mostly synthesized compounds exhibited peak at 11.19–12.65 ppm due to the presence of –NH of the indole moiety. The signals of the aromatic protons have been observed in the range of *δ* 6.37–8.58 ppm. The protons of the ethoxy group in **DS-2** produced a classic triplet-quartet signal pattern at *δ* 1.27 ppm and 4.13 ppm respectively. The proton signal of the methylene group as in the case of **DS-1** appeared as a singlet at 4.23 ppm. The proton of the carboxyl group appeared in the range of *δ* 10.24–11.59 ppm. The protons of the saturated carbons of the cyclohexenyl ring appeared as two multiplets at *δ* 2.73 (4H, 2CH_2_) and *δ* 1.85 (4H, 2CH_2_). The proton correspond to the sulphonamide (–SO_2_NH–) moiety in **DS-11** and **DS-19** appeared in the range of 5.91–5.98 ppm. The protons of methyl group were observed in the range of 1.80–2.31 ppm. The carbon signals of the aromatic carbons in ^13^C NMR spectrum of **DS-1** to **DS-21** were observed between 108 and 159 ppm. The ^13^C NMR peaks at 165–177 ppm accounted for the carbonyl group. The carbon of the imine group was observed between 160 and 165 ppm. The ethoxy carbons appeared at the 61 and 14 ppm respectively. The peak in the range of 45–58 ppm represented the methylene carbons. The carbon signals of saturated carbons of the cylohexenyl ring appeared in the range of 20–28 ppm. The final confirmation of the synthesized compounds was done by mass spectroscopy. The % of C, O, N, H and S in the target compounds was found to be within the defined limits.

### Biological activity

#### Antimicrobial results

The antimicrobial evaluation was done for the synthesized indole derivatives (**DS-1** to **DS-21**) in terms of MIC and MBC values in µg/ml using standard antibacterial drugs (ciprofloxacin and cefotaxime) and antifungal drug (fluconazole). The results of antimicrobial evaluation has been presented in Tables 1 and 2 respectively. The most of the indole derivatives had shown the highest activity against Gram-negative bacteria particularly *E. coli* and *K. pneumonia* with MIC ranges from 1.95 to 7.81 µg/ml comparable with the standard drugs. The derivative **DS-14** having substituted *p*-carboxy phenyl ring attached with the indole diazenyl scaffold was found most active against *E. coli, S. enterica* and *K. pneumonia* with MIC of 1.95–3.90 µg/ml. The derivatives **DS-6**, **DS-13**, **DS-14**, **DS-20**, and **DS-21** not only acted as bacteriostatic agents but also as bactericidal agents against *E. coli* by exhibiting low MBC values (3.90–7.81 µg/ml) in comparison to the standard drugs (15.62–31.25 µg/ml). Similarly, the derivatives **DS-1**, **DS-6**, **DS-10**, **DS-14**, and **DS-21** also acted as bactericidal agents against *K. pneumonia* with MBC values (3.90–7.81 µg/ml) in comparison with the standard drugs. The derivatives **DS-2** and **DS-3** had shown moderate activity against fungal strain *A. fumigatus* having MIC of 15.62 µg/ml as compared to the standard drug fluconazole (MIC = 7.81 µg/ml). All of the synthesized derivatives had shown less activity against tested Gram-positive bacteria (*S. aureus* and *B. subtilis)* and fungal strain (*A. niger)* with MIC > 10 µg/ml. These results clearly indicated that Gram negative bacteria were more susceptible to the synthetic indole derivatives.

**Table 1:** MIC in µg/ml of synthesized indole hybridized diazenyl derivatives

Compd.	*E. coli*	*S. aureus*	*S. enterica*	*B. subtilis*	*K. pneumonia*	*A. niger*	*A. fumigatus*
MIC (µg/ml)							
**DS-1**	15.62	62.5	62.5	62.5	3.90	125	62.5
**DS-2**	15.62	62.5	15.62	31.25	31.25	62.5	15.62
**DS-3**	3.90	62.5	31.25	31.25	1.95	62.5	15.62
**DS-5**	1.95	125	31.25	62.5	3.90	62.5	125
**DS-6**	1.95	31.25	31.25	31.25	7.81	62.5	31.25
**DS-7**	7.81	62.5	31.25	125	3.90	62.5	62.5
**DS-10**	1.95	62.5	31.25	62.5	3.90	62.5	125
**DS-11**	3.90	31.25	62.5	31.25	15.62	125	62.5
**DS-13**	7.81	62.5	62.5	62.5	3.90	62.5	31.25
**DS-14**	3.90	62.5	1.95	31.25	1.95	62.5	31.25
**DS-15**	15.62	125	31.25	31.25	3.90	125	62.5
**DS-16**	15.62	62.5	62.5	31.25	7.81	62.5	31.25
**DS-17**	3.90	62.5	62.5	125	7.81	62.5	31.25
**DS-19**	7.81	31.25	62.5	31.25	15.26	31.25	31.25
**DS-20**	1.95	125	15.62	125	3.90	62.5	62.5
**DS-21**	3.90	62.5	31.25	31.25	3.90	125	125
**CFT**	1.95	15.62	15.62	15.62	1.95	–	–
**CPR**	3.90	1.95	15.62	7.81	7.81	–	–
**FLU**	–	–	–	–	–	31.25	7.81

*CFT* cefotaxime, *CPR* ciprofloxacin, *FLU* fluconazole

**Table 2:** MBC/MFC in µg/ml of synthesized indole hybridized diazenyl derivatives (**DS1**–**DS21**)

Compound	*E. coli*	*S. aureus*	*S. enterica*	*B. subtilis*	*K. pneumonia*	*A. niger*	*A. fumigatus*
MBC/MFC (µg/ml)							
**DS-1**	125	125	62.5	125	7.81	125	125
**DS-2**	16.25	125	15.62	125	62.5	125	62.5
**DS-3**	31.25	62.5	31.25	62.5	31.25	125	62.5
**DS-5**	31.25	125	62.5	125	15.62	125	125
**DS-6**	3.90	31.25	31.25	62.5	7.81	125	125
**DS-7**	31.25	125	31.25	125	16.25	125	125
**DS-10**	31.25	125	62.5	125	7.81	125	125
**DS-11**	62.5	62.5	125	125	31.25	125	125
**DS-13**	3.90	62.5	62.5	125	15.62	125	125
**DS-14**	7.81	62.5	7.81	62.5	3.90	125	125
**DS-15**	125	31.25	31.25	62.5	31.25	125	125
**DS-16**	62.5	125	62.5	125	31.25	125	125
**DS-17**	15.62	62.5	62.5	62.5	15.62	125	125
**DS-19**	62.5	31.25	62.5	125	15.62	125	125
**DS-20**	7.81	62.5	31.25	125	15.62	125	125
**DS-21**	7.81	62.5	31.25	62.5	7.81	125	125
**CFT**	15.62	31.25	31.25	15.62	3.90	–	–
**CPR**	31.25	62.5	31.25	31.25	7.81	–	–
**FLU**	–	–	–	–	–	125	125

*CFT* cefotaxime, *CPR* ciprofloxacin, *FLU* fluconazole

From the SAR studies it was elucidated that

- The indole diazenyl scaffold is essential for activity against Gram-negative bacteria particularly *E. coli* and *K. pneumonia.*
- The derivative (**DS-14**) with substituted phenyl ring having carboxy group at the *para* position showed remarkable activity against tested Gram-negative bacteria.
- The derivative **DS-1** (with substituted furfuryl ring attached with the indole diazenyl scaffold) exhibited more activity against *K. pneumonia* as compared to *E. coli*.
- The derivatives **DS-6** (substituted pyrazole ring), **DS-20** (substituted ethylaminonapthyl ring) and **DS-21** (having substituted benzothiazolyl ring) acted as bactericidal agents against *K. pneumonia* and *E. coli*.
- The derivatives **DS-2** (substituted tetrahydrobenzothiophene ring) and **DS-3** (substituted thiadiazole ring) exhibited activity only against fungal strain *A. fumigatus*.
- The compounds **DS-11** and **DS-19** with substituted sulfa drugs (sulfapyridine and sulfadiazine) exhibited activity only against *E. coli* and acted as bacteriostatic agents.
- The most of the synthesized compounds were found to inactive against tested Gram-positive bacterial strains and fungal strain *A. niger*.

#### Anticancer results

**Fig. 3:**
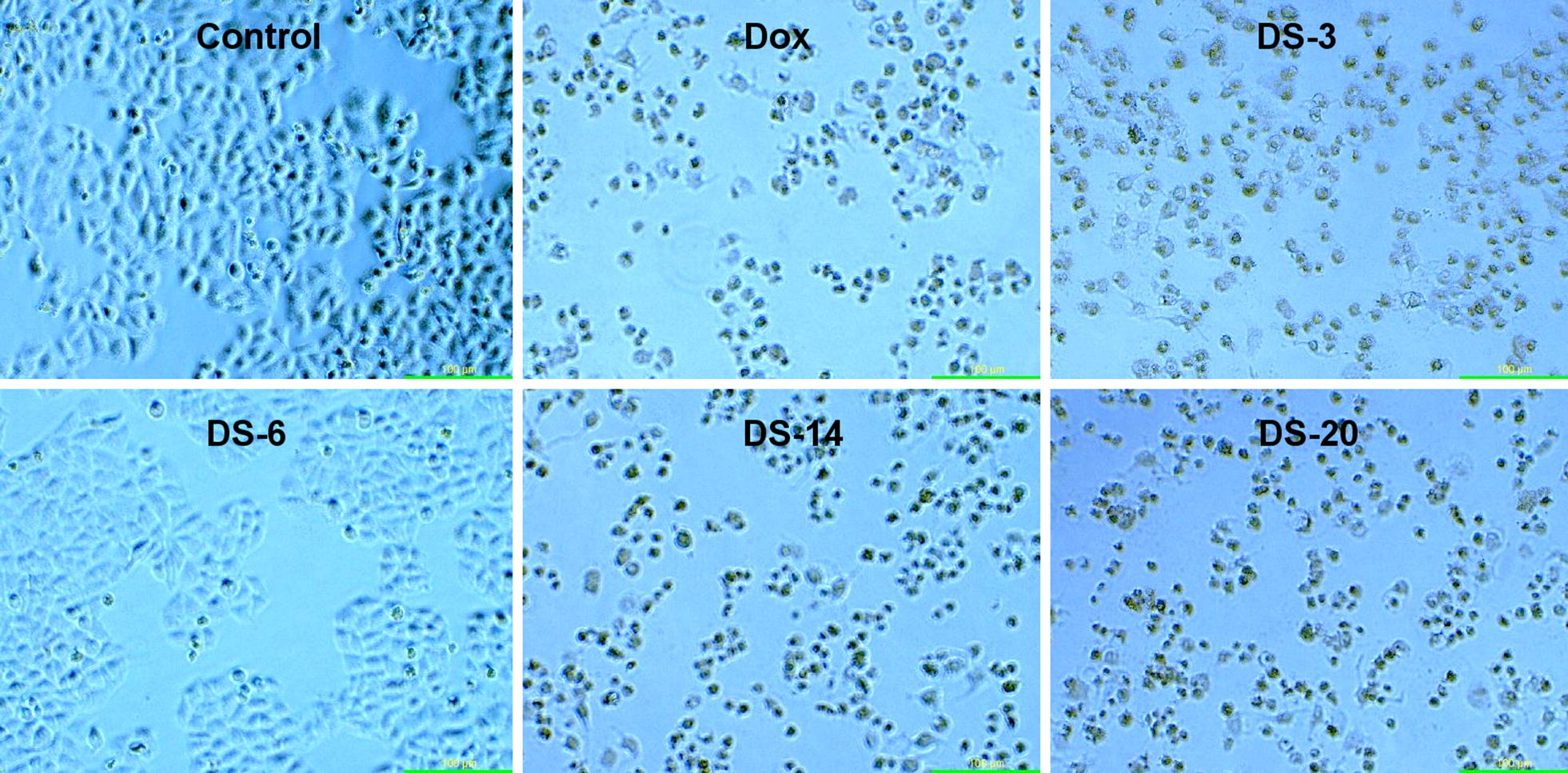
Morphological characterization of control, standard and various test compounds against MDAMB 231 cell line at 50 µg/ml using inverted phase microscope (Biolink) after 48 h

The selected compounds **DS-2**, **DS-3**, **DS-6**, **DS-10**, **DS-14**, **DS-20** and **DS-21** have been evaluated for cytotoxicity against human lung carcinoma cell line (HCT-116), breast cancer cell line (MDA MB231), leukemic cancer cell line (K562), and normal cell line (HEK293) by MTT assay using doxorubicin as the standard drug. The results of anticancer screening have been presented in Table 3. The IC_50_ was calculated from the survival curve plots which were drawn between % cell viability and concentration of the compounds. The experiment was performed in triplicate. The different cells treated with different concentrations of the test derivatives were also observed under inverted phase microscope (Biolink) at 48 h for various morphological changes like density of cells, shape of the cells, and any signs of shrinkage. The derivatives **DS-2**, **DS-3**, **DS-14**, **DS-20** and **DS-21** have shown cytotoxicity towards breast cancer cell line (MDA MB 231) with IC_50_ in the range of 20–60 µg/ml as compared to the standard drug doxorubicin (IC_50_ = 3 µg/ml). In Fig. 3, it is evident that the test derivatives have reduced the number and clumping of MDA MB 231 cells. The higher concentrations of the test derivatives have significantly reduced the number of MDA MB231 cells. The derivatives **DS-14** and **DS-20** had also shown moderate activity against human colon carcinoma cell line (HCT-116) with MIC of 54–67 µg/ml. None of the tested derivative had shown significant activity towards the leukemic cancer cell line (K562). The selected compounds were also evaluated for their possible cytotoxicity in human embryonic kidney cells (HEK-293) by employing MTT assay. The assay results suggested that these compounds did not significantly affect the growth of normal kidney cells (As most of the compounds IC_50_ values are > 100). Hence, these compounds revealed their safety for the normal cells and the compounds **DS-14** and **DS-20** can be taken as lead compounds for further development of more potential agents for breast cancer.

**Table 3:** IC_50_ values (in µg/ml) of indole diazenyl derivatives against various cell lines

Compound	HCT 116^a^	MDA MB 231^b^	K562^c^	HEK-293^d^
IC_50_ (µg/ml)				
**DS-2**	116.57 ± 4.28	57.63 ± 3.68	140.05 ± 4.40	280.24. ± 1.74
**DS-3**	110.03 ± 2.55	45.96 ± 1.83	146.08 ± 2.11	311.17 ± 1.76
**DS-6**	139.19 ± 4.29	118.73 ± 1.92	187.76 ± 1.94	257.84 ± 0.57
**DS-10**	204.93 ± 1.62	129.66 ± 3.51	198.55 ± 3.13	274.12 ± 4.58
**DS-14**	54.03 ± 1.96	19.10 ± 1.43	112.26 ± 3.64	244.15 ± 3.78
**DS-20**	66.66 ± 1.56	20.95 ± 1.93	135.80 ± 4.74	232.69 ± 4.68
**DS-21**	124.80 ± 1.33	49.30 ± 4.15	157.95 ± 3.73	141.92 ± 3.25
**DOX**	3.37 ± 0.37	3.08 ± 0.95	2.62 ± 0.65	NT

IC_50_ = 50% Inhibitory concentration after 48 h of drug treatment

*DOX* doxorubicin, *NT* not tested

^a^Colon cancer

^b^Breast cancer

^c^Leukemia

^d^Normal cells

### Molecular docking

Bacterial DNA gyrase is the key enzyme and the target for the development of the newer antibacterial drugs [32]. DNA gyrase consists of 2 subunits-subunit A and subunit B. Subunit A nicks the dsDNA, subunit B introduces −ve supercoils and then subunit A reseals the ends. [33]. Further the functions of the DNA gyrase in humans are performed by the group of topoisomerase enzymes. The natural product (e.g., coumarin) and synthetic compounds (e.g., quinolone) are well known inhibitors of bacterial gyrase [34]. The coumarin antibiotics, novobiocin and its dimer coumermycin A1 are characteristic examples, which inhibit the subunit B of DNA gyrase. But the use of these inhibitors has been restricted due to emergence of drug resistance [35]. Therefore, targeting subunit B might produce some novel DNA gyrase inhibitors.

**Fig. 4:**
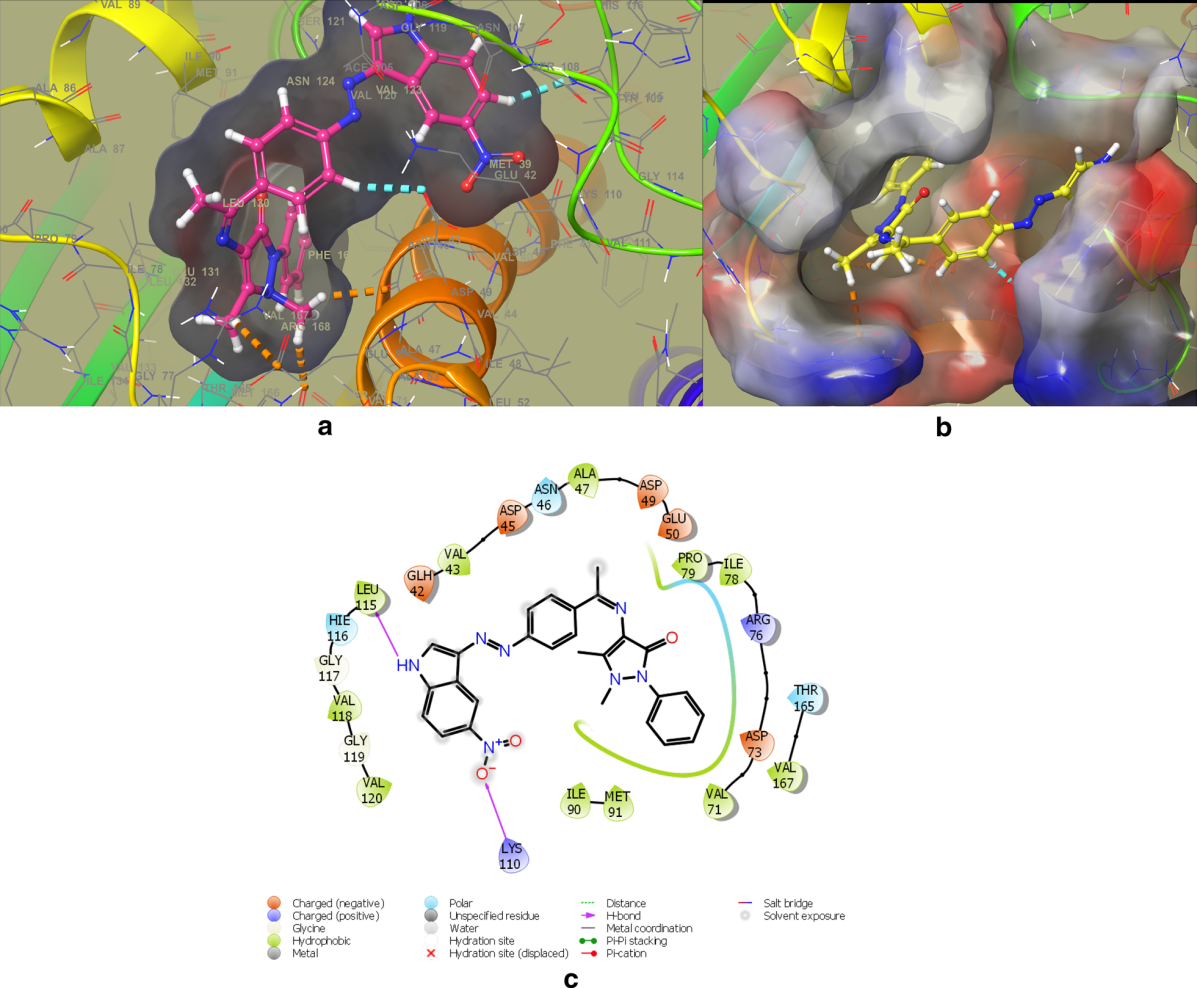
**a** Best docked pose of **DS-6** with GyrB (4KFG), **b** surface binding of **DS-6** with 4KFG, **c** ligand Interaction diagram of **DS-6** with 4KFG

**Fig. 5:**
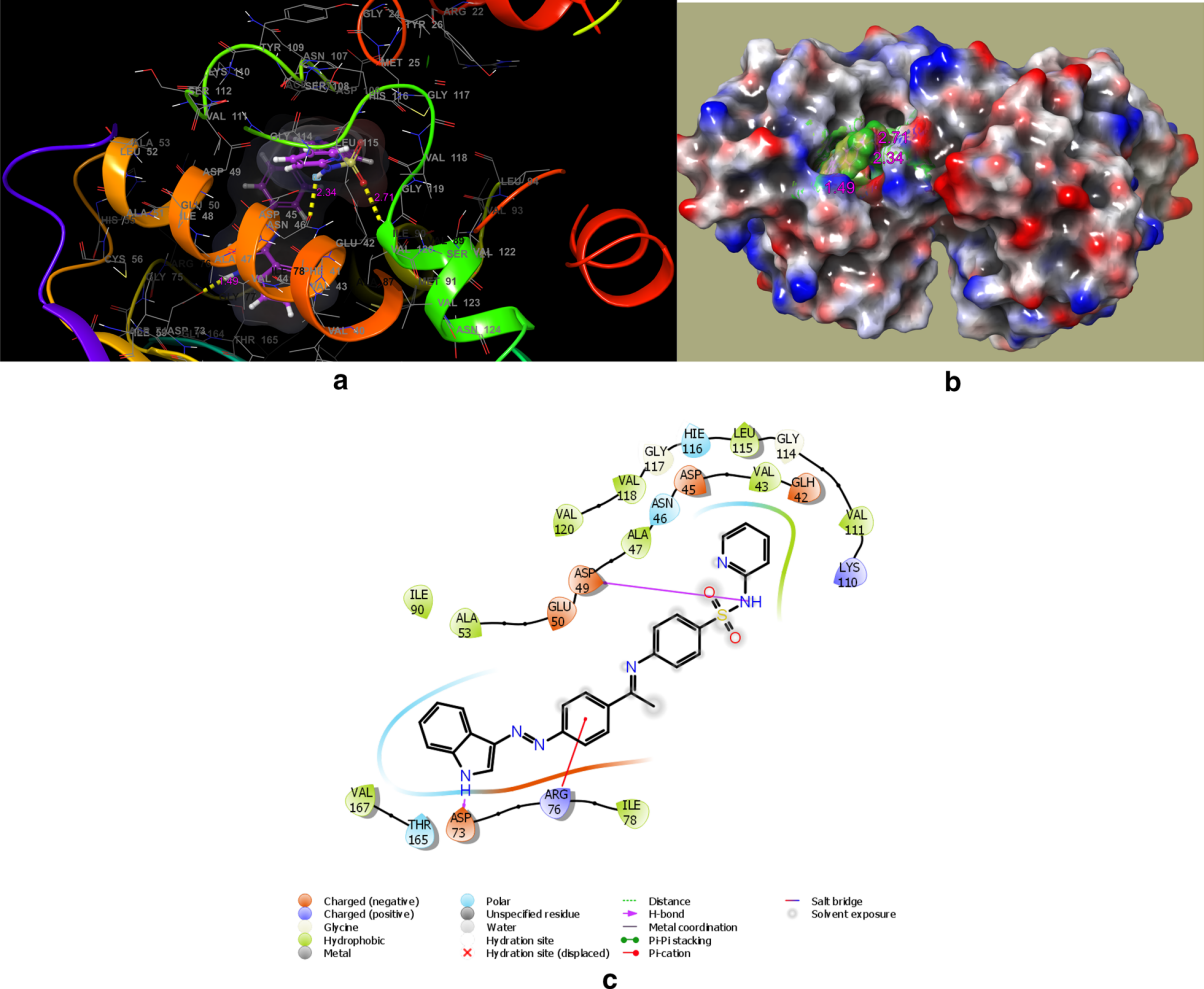
**a** Best docked pose of **DS-11** with GyrB (4KFG), **b** surface binding of **DS-11** with 4KFG, **c** ligand interaction diagram of **DS-11** with 4KFG

**Fig. 6:**
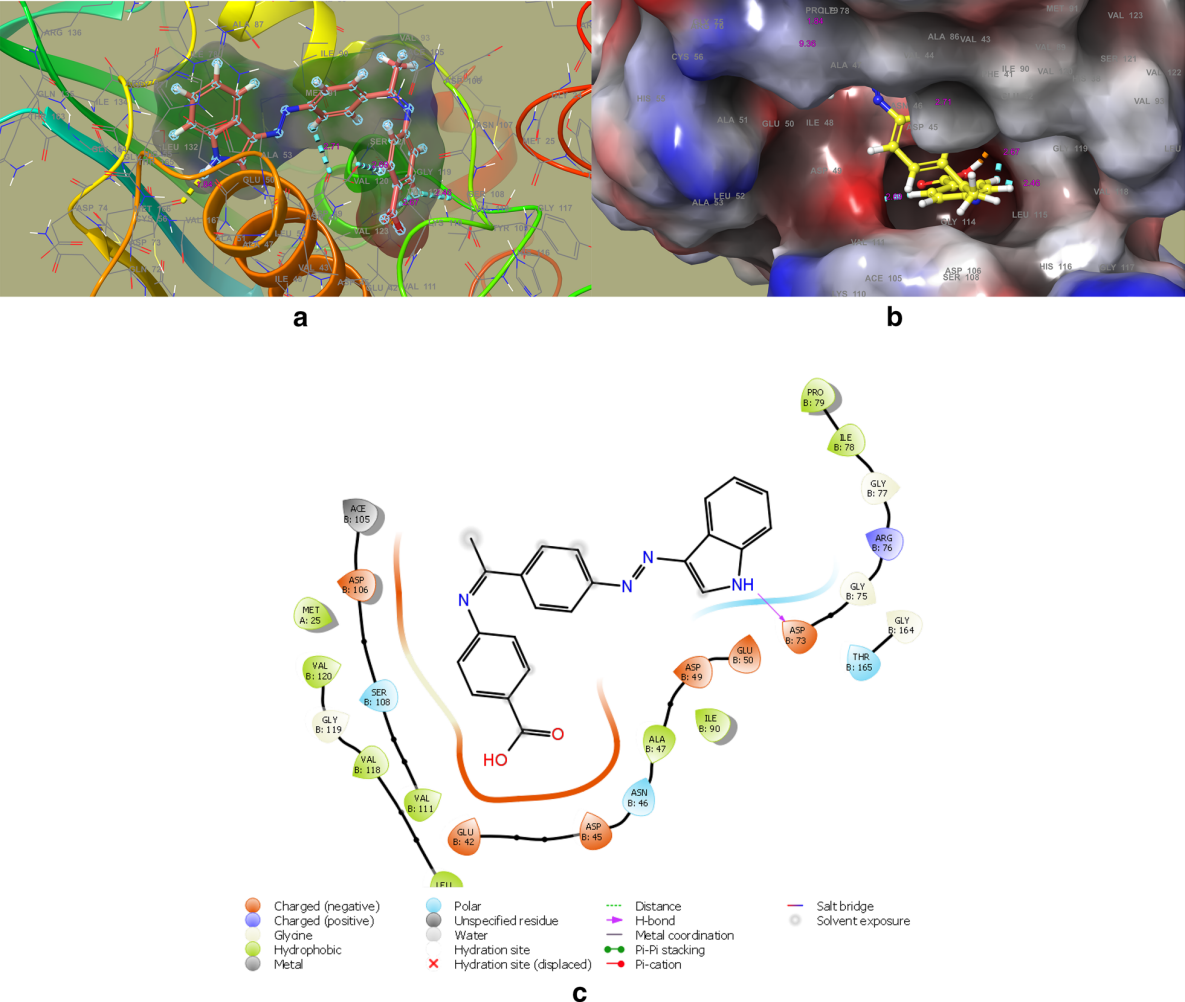
**a** Best docked pose of **DS-14** with GyrB (4KFG), **b** surface binding of **DS-14** with 4KFG, **c** ligand interaction diagram of **DS-14** with 4KFG

**Fig. 7:**
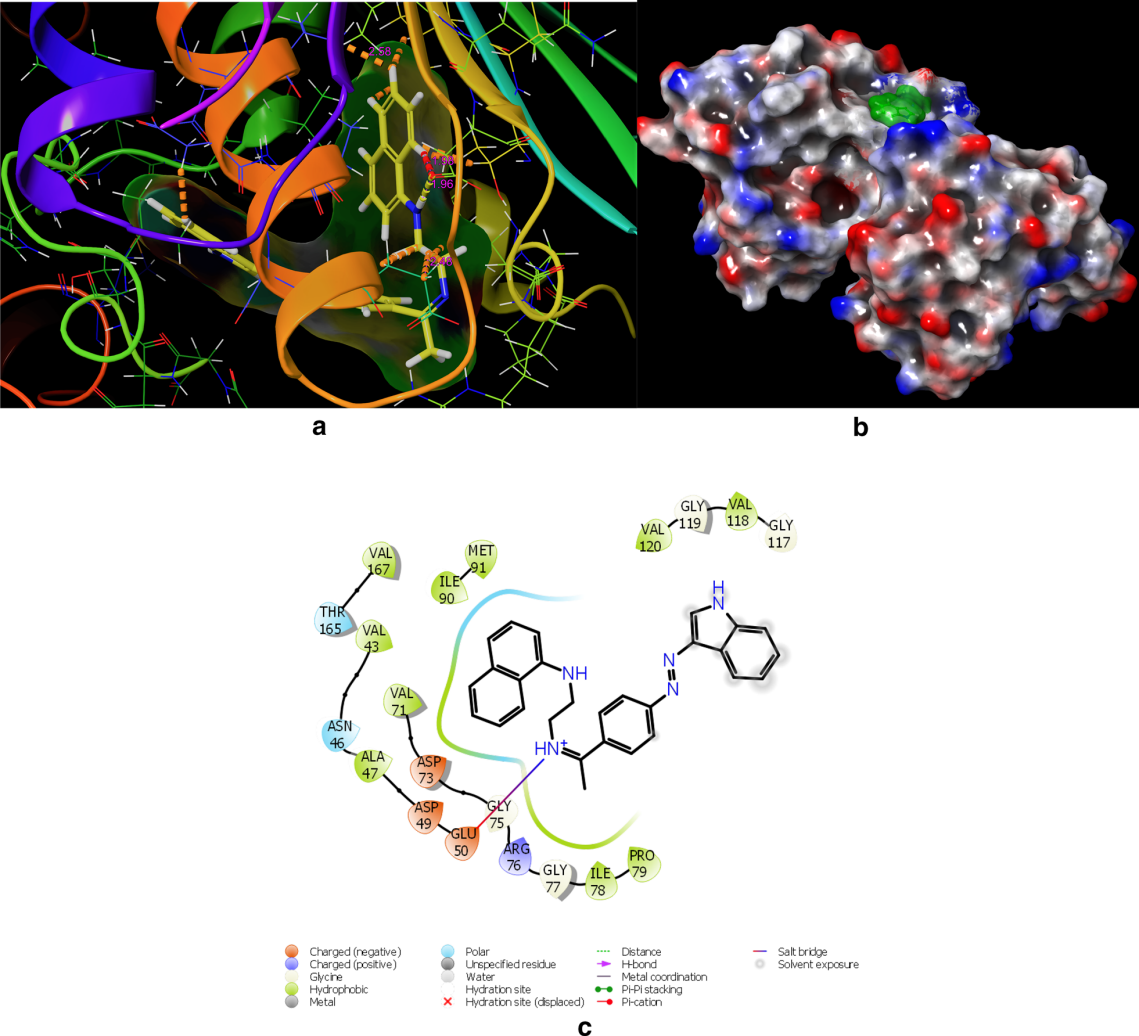
**a** Best docked pose of **DS-20** with GyrB (4KFG), **b** surface binding of **DS-20** with 4KFG, **c** Ligand interaction diagram of **DS-20** with 4KFG

In the DNA gyrase subunit B, there is an ATP binding pocket, where ATP bind and on hydrolysis activate the enzyme for the further function. Thus targeting this site might be effective to produce novel DNA gyrase inhibitors. In an effort to find out the new inhibitors of the GyrB subunit ATP binding site and minimize target-based resistance, the target compounds were docked on the ATPase binding site of GyrB from *E. coli* (PDB:4KFG). The molecular docking study was carried out on GLIDE docking program and the results were analyzed based on the docking score, glide energy, glide energy model obtained from GLIDE and presented in Table 4. The predicted docking poses were visually inspected and interactions with the binding pocket residues were analyzed using the ligand-interactions diagrams. The docking results showed that the studied compounds can be accommodated in the binding pocket of GyrB subunit with a comparable orientation to the one observed in the co-crystallised ligand covalent adduct in the reported crystal structure (4KFG). The best docked pose for the highest active compounds **DS-6**, **DS-11**, **DS-14**, **DS-20** have been presented in Figs. 4, 5, 6, and 7 respectively. The docking scores were demonstrated in terms of negative energy; the lower the binding energy, best would be the binding affinity. The most of the compounds have shown good docking scores and low binding energies as compared to the standard drug novobiocin and comparable scores with the ciprofloxacin. The highest docking score (− 5.921) was observed for **DS-20** followed by **DS-11** (− 5.469) and **DS-14** (− 5.123) in comparison to the standard drugs novobiocin (− 3.455) and ciprofloxacin (− 5.071).

**Table 4:** Glide docking scores of Indole diazenyl derivatives (**DS1**–**DS21**) against GyrB subunit (4KFG)

S. no	Glide Emodel	Glide energy	Glide evdw	Docking score
**DS-1**	− 61.121	− 45.842	− 44.011	− 4.332
**DS-2**	− 84.18	− 56.696	− 51.197	− 4.356
**DS-3**	− 106.573	− 64.868	− 62.844	− 3.182
**DS-6**	− 81.579	− 59.847	− 56.86	− 5.255
**DS-7**	− 65.006	− 49.231	− 44.718	− 3.981
**DS-10**	− 72.221	− 49.484	− 47.959	− 4.477
**DS-11**	− 89.575	− 64.032	− 53.014	− 5.469
**DS-13**	− 67.971	− 54.886	− 49.797	− 4.022
**DS-14**	− 79.274	− 54.871	− 47.968	− 5.123
**DS-15**	− 90.979	− 55.621	− 59.366	0.647
**DS-16**	− 78.016	− 53.488	− 52.037	− 4.902
**DS-17**	− 65.955	− 46.798	− 44.872	− 4.119
**DS-19**	− 88.711	− 60.349	− 51.526	− 5.411
**DS-20**	− 83.895	− 56.311	− 52.249	− 5.921
**DS-21**	− 89.289	− 59.604	− 57.047	− 3.864
**CFT**	− 83.319	− 57.891	− 50.792	− 6.459
**CPR**	− 51.008	− 41.768	− 39.321	− 5.071
**NOV**	− 60.31	− 50.268	− 37.426	− 3.455

*CFT* cefotaxime, *CPR* ciprofloxacin, *NOV* novobiocin

The synthesized indole diazenyl derivatives (**DS-1** to **DS-21**) showed that in most of the active inhibitors the interactions were dominated by the hydrogen bonding (1.34–1.98 Å) due to the presence of –NH of the indole moiety with the essential Asp73 residue of the binding site (same interaction as in case of co-crystallised ligand). The most of the active inhibitors exhibited hydrogen bonding network with Asn46, Asp73, Arg76, Val120, the key residues belonging to the catalytic domain of the GyrB enzyme amino acid stretch. The compounds with substituted nitro group exhibited two salt bridges through nitrogen and oxygen of nitro group with the key residues Arg76 (4.62 Å) and Glu50 (4.64 Å). The active compound **DS-11** exhibited three hydrogen bonding interactions: –NH– of the indole moiety with the Asp73 residue (1.49 Å), –S=O moiety with the Val120 residue (2.71 Å), through sulfamoyl –NH moiety with the Asn46 residue (2.34 Å). Another active compound **DS-6** did not display any H-bonding interaction with the key amino acid residues. The main interactions dominated in this compound are electrostatic and hydrophobic interactions at the several key residues. The interacting amino acids at the active site with the different test compounds have been presented in Table 5. Overall the docking results validated the antimicrobial activity and these indole diazenyl derivatives can be developed as lead compounds as DNA gyrase inhibitors.

**Table 5:** The most active derivatives interaction with the key amino acid residues of the ATP binding pocket of GyrB subunit (PDB ID: 4KFG) from *E. coli*

Compound	Interacting residues at the active site
**DS-6**	Pro79, Ile78, Gly77, Arg76, Asp73, Ile90, Met91, Glu50, Asp49, Ala47, Asn46, Asp45, Val43, Glu42, Val120, Val118, His116, Leu 115, Val111, Ser108, Asp106
**DS-10**	Asn46, Ala47, Asp49, Glu50, Pro79, Ile78, Arg76, Asp73, Thr165, Ile 90, Arg76, Asp73, Thr165, Ile 90, Asp106, Asn 107, Ser108
**DS-11**	Val120, Gly119, Glu42, Val43, Asp45, Asn46, Ala47, Asp49, Glu50, Ser108, Asp73, Arg76
**DS-14**	Pro79, Ile78, Arg76, Asp73, Glu50, Asp49, Ala47, Asn46, Asp45, Glu42, Val111, Ser108, Asp106
**DS-16**	Pro79, Ile78, Arg76, Asp73, Glu50, Asp49, Ala47, Asn46, Asp45, Glu42, Val111, Ser108, Asn107, Asp106
**DS-19**	Asp73, Val71, Glu50, Asp49, Ala47, Asn46, Asp45, Val43, Glu42, Leu115, His116, Val118, Gly119, Val120, Ser108, Asp106
**DS-20**	Pro79, Ile78, Gly77, Arg76, Asp73, Gln72, Val71, Glu50, Ala47, Asn46, Asp45, Val43, Glu42
**DS-21**	Pro79, Ile78, Arg76, Asp73, Glu50, Asp49, Asp73, Ala47, Asn46, Val120, Asp45, Val118
**CPR**	Ile90, Val120, Leu132, Val167, Val43, Asn46, Ala47, Thr165, Val71, Asp73, Arg76, Ile78, Pro79
**NOV**	Ile90, Ala53, Glu50, Asp49, Ala47, Asn46, Lys110, Thr165, Asp73, Arg76, Ile78, Pro79

*CPR* ciprofloxacin, *NOV* novobiocin

### ADME results

Mostly drugs failed during the clinical development due to the ADME/Tox deficiencies. So, the virtual screening should not be limited to improve selectivity and optimize binding affinity; but the pharmacokinetic parameters should also be involved as significant filters in virtual screening [36]. Over-all 44 descriptors and pharmaceutically pertinent properties of substituted indole diazenyl analogs were investigated using Qikprop (QikProp, version 3.5, Schrödinger) in comparison with those of 95% of known drugs [37]. Some of the important descriptors essential for envisaging the drug like properties of molecules are reported in Table 6. The most of the synthesized compounds have followed the Lipinski’s rules: molecular weight < 500 Da, octanol/water partition coefficient (QPlogPo/w) < 5, hydrogen bond acceptor < 10 and donor < 5. The % oral absorption was found in the range of 80–100% in most of the derivatives.

**Table 6:** ADME properties of indole diazenyl Schiff bases (**DS1**–**DS21**) by Qikprop module of Schrodinger

Comp.	MW	Donor HB	Acceptor HB	(Log Po/w)	(QPlogS)	(QPPMDCK)	(QPlogBB)	Rule of Five	% Oral absorption
**DS-1**	342.399	1.00	3.50	4.888	− 5.776	864.120	− 0.617	0	100
**DS-2**	470.588	1.00	5.00	6.083	− 7.814	564.131	− 1.016	1	100
**DS-3**	546.947	1.00	7.00	4.160	− 7.620	8.456	− 3.210	2	41.89
**DS-5**	512.502	1.00	7.00	3.729	− 7.238	3.533	− 3.464	2	38.89
**DS-6**	493.524	1.00	8.00	4.595	− 7.214	71.086	− 1.885	0	93.59
**DS-7**	374.462	1.00	5.00	4.211	− 5.864	343.064	− 1.110	0	100
**DS-10**	452.299	1.00	4.00	5.219	− 7.051	386.353	− 1.300	1	85.55
**DS-11**	494.570	2.00	8.500	4.422	− 6.748	85.060	− 1.945	0	93.76
**DS-13**	417.854	1.00	4.00	4.320	− 4.299	493.198	− 0.688	0	100
**DS-14**	382.421	2.00	5.00	3.949	− 3.722	47.744	− 0.975	0	85.21
**DS-15**	504.519	2.00	9.00	2.377	− 3.734	13.240	− 2.063	2	42.512
**DS-16**	401.399	1.00	4.00	4.627	− 6.447	148.752	− 1.521	0	94.824
**DS-17**	340.387	1.00	5.00	3.227	− 3.492	353.077	− 0.687	0	100.000
**DS-19**	495.558	2.00	9.50	3.858	− 6.342	65.284	− 2.073	0	88.565
**DS-20**	431.539	2.00	4.00	6.504	− 7.473	997.487	− 0.729	1	100.00
**DS-21**	440.478	1.00	5.50	4.377	− 6.667	76.004	− 1.861	0	89.828
**CFT**	455.460	3.25	12.95	0.586	− 7.254	1.758	− 3.778	1	21.057
**CPR**	331.346	1.00	6.00	0.280	− 4.829	10.642	− 0.643	0	48.759
**NOV**	612.632	5.25	13.15	2.612	− 3.791	3.490	− 4.055	3	21.432

*CFT* cefotaxime, *CPR* ciprofloxacin, *NOV* novobiocin, *MW* molecular weight, *DonorHB* hydrogen bond donor, *AcceptorHB* hydrogen bond acceptor, *P*_*O/W*_ partition coefficient in oil and water, *QPlogS* aqueous solubility, *QPPMDCK* apparent MDCK cell permeability, *QPlogBB* brain/blood partition coefficient

## Experimental

### Materials and methods

The required synthetic chemicals were purchased from Merck Chemicals (India) and utilized without further purification. The reagents for the antimicrobial evaluation and for the cytotoxicity study were procured from Hi-Media Laboratories (India). The microbial strains were acquired from Institute of Microbial Technology and Genebank (IMTECH), Chandigarh. The IR spectra was recorded on the Bruker 12060280 FTIR spectrophotometer using KBr pellet method. The Bruker Avance II 400 NMR spectrometer was used for carried out the NMR spectroscopy (^1^H NMR and ^13^C NMR), for the synthesized derivatives in deuterated DMSO solvent. The structures of the synthesized derivatives were confirmed by mass spectra, taken on the Advion expression CMS, USA mass spectrometer with APCI mode as the ion source.

### Synthetic procedure for indole diazenyl Schiff bases

The *p*-aminoacetophenone (0.01 mol, 1.35 g) was dissolved in 10 ml solution of 0.2 N HCl followed by addition of 5 ml solution of sodium nitrite (0.01 mol) at 0–5 °C to complete the diazotization process. The indole/5-nitroindole (0.01 mol) was solubilized in 20 ml of acetic/propionic acid (8:2) mixture and cooled at 0 °C. The diazotized solution was added to the indole derivative gradually at 0 °C over a period of 10–15 min followed by stirring for 1–2 h in the cold conditions. Afterwards saturated sodium carbonate solution (20–25 ml) was added to precipitate the azo dye (**1**, **2**) with continuous stirring for further half an hour [38, 39]. The precipitated azo dye was filtered, vacuum dried and recrystallized from ethanol and used further for the synthesis of indole diazenyl Schiff bases. In 250 ml round bottom flask, indole azo dye (0.001 M) was refluxed with aromatic/heteroaromatic amine (0.001 M) in the presence of ethanol and catalytic amount of acid on a heating mantle. The refluxing was continued for 7–8 h until the reaction completion was confirmed by TLC. The reaction volume was concentrated and kept at 7–8 °C for 24 h for the precipitation of Schiff bases. The synthesized Schiff bases were purified by recrystallization and column chromatography using ethyl acetate: methanol (70:30) on silica gel 100–200 mesh size [27, 40].

### Analytical data

1-[4-[(*E*)-1*H*-Indol-3-ylazo]phenyl]ethanone (**1**): MF: C_16_H_13_N_3_O; 263.29; Maroon color; Yield: 92%; mp: 90–95 °C: IR (KBr, cm^−1^) ν_max_: 3217, 2921, 1668, 1592, 1460, 1424, 1358, 1266, 1215, 1169, 1053, 1014, 959, 837, 748, 624, 594, 446; ^1^H NMR (400 MHz, DMSO-d_6_) δ: 11.96 (s, 1H, –NH– of indole), 9.01 (s, 1H, –CH(2) of indole), 8.16 (d, *J *= 2.4 Hz, 1H, –CH(4) of indole), 8.01 (d, *J*_*1*_= 8.8 Hz, 2H, ArH), 7.51–7.76 (m, 2H, –CH(6,7) of indole), 7.32 (d, *J *= 8.8 Hz, 2H, ArH), 7.21–7.26 (m, 1H, –CH(5) of indole), 2.18 (s, 3H, CH_3_); ^13^C NMR (100 MHz, DMSO-d_6_) δ: 198.2, 148.8, 140.1, 138.7, 133.7, 128.5, 124.8, 122.9, 117.6, 117.4, 116.7, 115.5, 113.4, 112.3, 26.2.

1-[4-[(*E*)-(5-Nitro-1*H*-indol-3-yl)azo]phenyl]ethanone (**2**): MF: C_16_H_12_N_4_O_3_; 308.29; Orange color; Yield: 69%; mp: 65–70 °C; IR (KBr, cm^−1^) ν_max_: 3328, 2925, 1707, 1669, 1617, 1517, 1468, 1426, 1329, 1167, 1108, 1065, 894, 826, 780, 743, 682, 588, 540, 432; ^1^H NMR (400 MHz, DMSO-d_6_) δ: 12.01 (s, 1H, –NH– of indole), 9.97 (s, 1H, –CH(2) of indole), 8.35–8.47 (m, 1H, –CH(4) of indole), 8.22 (d, *J *= 2.4 Hz, 1H, –CH(7) of indole), 7.98 (dd, *J*_*1*_= 8.8 Hz, *J*_*2*_= 2.4 Hz, 1H, –CH(6) of indole), 7.56–7.63 (m, 2H, ArH), 7.11 (d, *J *= 8.8 Hz, 2H, ArH), 2.09 (s, 3H, CH_3_); ^13^C NMR (100 MHz, DMSO-d_6_) δ: 198.6, 162.9, 141.0, 140.8, 138.81, 133.8, 128.5, 124.8, 122.8, 117.5, 117.4, 116.7, 115.1, 113.2, 112.3, 26.3.

(*E*)-*N*-(2-Furylmethyl)-1-[4-[(*E*)-1*H*-indol-3-ylazo]phenyl]ethanimine (**DS-1**): Dark Maroon; Yield: 75%; R_f_ = 0.57 (ethyl acetate/methanol 8:2); mp: 315–320 °C; IR (KBr, cm^−1^) ν_max:_ 3297, 3055, 2922, 1641, 1596, 1401, 1355, 1264, 1158, 1011, 958, 884, 837, 744, 593, 498, 431; ^1^H NMR (400 MHz, DMSO-d_6_) δ: 11.19 (s, 1H, –NH– of indole), 9.02 (s, 1H, –CH(2) of indole), 8.38 (d, *J *= 8.0 Hz, 1H, –CH (4) of indole), 8.09 (d, *J *= 8.4 Hz, 2H, ArH), 7.8–8.02 (m, 1H, –CH(7) of indole), 7.63 (d, *J *= 8.4 Hz, 2H, ArH), 7.36–7.59 (m, 2H, –CH(5,6) of indole), 7.02 (d, *J *= 7.2 Hz, 1H, –CH(5) of furan), 6.84 (d, *J *= 7.2 Hz, 1H, –CH(4) of furan), 6.37 (d, *J *= 7.2 Hz, 1H, –CH(3) of furan), 4.23 (s, 2H, CH_2_), 1.89 (s, 3H, CH_3_); ^13^C NMR (100 MHz, DMSO-d_6_) δ: 170.2, 155.5, 152.2, 147.6, 140.9, 137.6, 135.2, 125.7, 126.5, 122.3, 121.9, 121.8, 121.0, 120.5, 117.3, 115.2, 109.1, 56.2, 23.5; APCI-MS m/z found for C_21_H_18_N_4_O: 342.39 (M^+^); Anal. calcd for C_21_H_18_N_4_O: C 73.67, H 5.30, N 16.36, O 4.67 found C 73.69, H 5.35, N 16.39, O 4.65.

Ethyl 2-((Z)-(1-(4-((Z)-(1*H*-indol-3-yl)diazenyl)phenyl)ethylidene)amino)-4,5,6,7-tetrahydrobenzo[*b*]thiophene-3-carboxylate (**DS-2**): Dark Maroon; Yield: 79%; mp: 260–265 °C; R_f_ = 0.49 (ethyl acetate/methanol 8:2); IR (KBr, cm^−1^) ν_max_: 3404, 3298, 3169, 2982, 2934, 2847, 1721, 1646, 1593, 1488, 1413, 1373, 1335, 1278, 1149, 1024, 967, 894, 836, 744, 601, 510, 469, 432; ^1^H NMR (400 MHz, DMSO-d_6_) δ: 11.29 (s, 1H, –NH– of indole), 8.92 (s, 1H, –CH(2) of indole), 8.10 (d, *J *= 8.8 Hz, 2H, ArH), 7.95 (d, *J *= 8.8 Hz, 2H, ArH), 7.41 (d, *J *= 7.6 Hz, 1H, –CH(4) of indole), 7.25–7.35 (m, 1H, –CH(7) of indole), 7.04–7.09 (m, 1H, –CH(5) of indole), 6.84 (d, *J *= 4.0 Hz, 1H, –CH(6) of indole), 4.13 (q, *J *= 9.6 Hz, 2H, –OCH_2_), 2.74–2.82 (m, 4H, CH_2_), 1.90 (s, 3H, CH_3_), 1.44–1.84 (m, 4H, CH_2_), 1.23 (t, *J *= 9.6 Hz, 3H, CH_3_); ^13^C NMR (100 MHz, DMSO-d_6_) δ: 175.3, 169.6, 160.6, 155.3, 142.2, 139.1, 137.5, 135.3, 132.5, 129.2, 127.5, 125.4, 123.6, 123.0, 122.2, 121.4, 120.2, 115.7, 61.8, 26.5, 26.3, 24.4, 23.6, 22.5, 14.3; APCI-MS m/z found for C_27_H_26_N_4_O_2_S: 470.18 (M^+^); Anal. calcd for C_27_H_26_N_4_O_2_S: C 68.91, H 5.57, N 11.91, O 6.80, S 6.81 found C 68.95, H 5.54, N 11.88, O 6.82.

(Z)-5-(2-Chloro-4-nitrophenyl)-*N*-(1-(4-((Z)-(5-nitro-1*H*-indol-3-yl)diazenyl)phenyl) ethylidene)-1,3,4-thiadiazol-2-amine (**DS-3**): Orange; Yield: 68%; mp: 155–160 °C; R_f_ = 0.61 (ethyl acetate/methanol 7:3); IR (KBr, cm^−1^) ν_max_: 3347, 3107, 2924, 1707, 1669, 1617, 1519, 1467, 1332, 1221, 1143, 1062, 963, 915, 827, 782, 740, 684, 656, 590, 535; ^1^H NMR (400 MHz, DMSO-d_6_) δ: 12.65 (s, 1H, –NH– of indole), 9.37 (s, 1H, –CH(2) of indole), 8.75 (d, *J *= 2.4 Hz, 1H, –CH(4) of indole), 8.62 (s, 1H, CH–C–Cl), 8.34 (dd, *J*_*1*_= 8.4 Hz, *J*_*2*_= 2.4 Hz, 1H, –CH=C–NO_2_), 8.24 (dd, *J*_*1*_= 8.4 Hz, *J*_*2*_= 2.8 Hz, 1H, –CH(6) of phenyl), 8.15 (dd, *J*_*1*_= 9.2 Hz, *J*_*2*_= 2.4 Hz, 1H, CH(6) of indole), 7.91 (d, *J *= 2.4 Hz, 1H, –CH(7) of indole), 7.55–7.75 (m, 4H, ArH), 2.07 (s, 3H, CH_3_); ^13^C NMR (100 MHz, DMSO-d_6_) δ: 180.2, 173.2, 155.3, 148.5, 139.7, 139.5, 138.5, 137.5, 135.2, 132.2, 130.6, 129.5, 125.5, 124.4, 121.8, 121.7, 121.0, 117.7, 114.3, 23.6; APCI-MS m/z found for C_24_H_15_ClN_8_O_4_S: 546.94 (M^+^); Anal. calcd for C_24_H_15_ClN_8_O_4_S: C 52.70, H 2.76, Cl 6.48, N 20.49, O 11.70 S 5.86 found C 52.72, H 2.78, N 20.53, O 11.73.

(Z)-*N*-(1-(4-((Z)-(5-Nitro-1*H*-indol-3-yl)diazenyl)phenyl)ethylidene)-5-(4-nitrophenyl)-1,3,4-thiadiazol-2-amine (**DS-5**): Orange; Yield: 57%; mp: 160–165 °C; R_f_ = 0.55 (ethyl acetate/methanol 7:3); IR (KBr, cm^−1^) ν_max_: 3359, 2923, 2854, 1831, 1706, 1617, 1518, 1467, 1332, 1219, 1169, 1112, 1062, 929, 850, 823, 783, 742, 686, 593, 533, 449, 430; ^1^H NMR (400 MHz, DMSO-d_6_) δ: 11.92 (s, 1H, –NH– of indole), 9.89 (s, 1H, –CH(2) of indole), 8.50 (s, 1H, –CH(4) of indole), 8.32–8.42 (m, 2H, ArH), 8.14–8.15 (m, 2H, ArH), 8.13 (d, *J *= 2.4 Hz, 1H, –CH(7) of indole), 7.87–7.94 (m, 2H, ArH), 7.45–7.56 (m, 2H, ArH), 7.03 (d, *J *= 9.2 Hz, 1H, ArH), 2.02 (s, 3H, CH_3_); ^13^C NMR (100 MHz, DMSO-d_6_) δ: 180.1, 170.1, 151.2, 147.2, 143.8, 139.9, 139.8, 136.9, 135.4, 130.5, 128.9, 128.6, 124.6, 124.4, 122.6, 121.8, 120.9, 117.9, 114.3, 49.3, 23.6; APCI-MS m/z found for C_24_H_16_N_8_O_4_S: 512.5 (M^+^); Anal. calcd for C_24_H_16_N_8_O_4_S: C 56.25, H 3.15, N 21.86, O 12.49, S 6.26 found C 56.28, H 3.14, N 21.89, O 12.52.

1,5-Dimethyl-4-((Z)-(1-(4-((Z)-(5-nitro-1*H*-indol-3-yl)diazenyl)phenyl)ethylidene) amino)-2-phenyl-1H-pyrazol-3(2*H*)-one (**DS-6**): Dark Yellow; Yield: 64%; R_f_ = 0.51 (ethyl acetate/methanol 8:2); mp: 170–175 °C; IR (KBr, cm^−1^) ν_max_: 3348, 3124, 2924, 1705, 1619, 1518, 1468, 1425, 1329, 1252, 1167, 1112, 1062, 927, 895, 823, 785, 741, 688, 656, 591, 538, 448; ^1^H NMR (400 MHz, DMSO-d_6_) δ: 11.99 (s, 1H, –NH of indole), 9.97 (s, 1H, –CH(2) of indole), 8.57 (d, *J *= 2.0 Hz, 1H, –CH(4) of indole), 8.43 (dd, *J*_1_ = 9.2 Hz, *J*_*2*_= 2.4 Hz, 1H, –CH(6) of indole), 8.37 (d, *J *= 2.4 Hz, 1H, –CH(7) of indole), 8.22 (d, *J *= 8.4 Hz, 2H, ArH), 7.98 (dd, *J*_1_ = 9.2 Hz, *J*_*2*_= 2.4 Hz, 1H, ArH), 7.58–7.63 (m, 2H, ArH), 7.11 (d, *J *= 9.2 Hz, 1H, ArH), 6.73–6.74 (m, 1H, ArH), 2.11 (s, 3H, CH_3_), 1.98 (s, 3H, CH_3_), 1.92 (s, 3H, CH_3_); ^13^C NMR (100 MHz, DMSO-d_6_) δ: 162.2, 161.9, 151.2, 147.5, 139.9, 139.8, 139.4, 135.5, 132.1, 130.5, 129.1, 126.8, 126.4, 124.6, 124.3, 124.2, 121.9, 120.9, 118.1, 114.3, 36.2, 22.6, 13.9; APCI-MS m/z found for C_27_H_23_N_7_O_3_: 493.52 (M^+^); Anal. calcd for C_27_H_23_N_7_O_3_: C 65.71, H 4.70, N 19.87, O 9.73 found C 65.75, H 4.67 N 19.89, O 9.69.

(Z)-*N*-(1-(4-((Z)-(1*H*-Indol-3-yl)diazenyl)phenyl)ethylidene)-5-ethyl-1,3,4-thiadiazol-2-amine (**DS-7**): Dark Maroon; Yield: 70%; mp: 145–150 °C; R_f_ = 0.47 (ethyl acetate/methanol 7:3); IR (KBr, cm^−1^) ν_max_: 3262, 3050, 2977, 2921, 2751, 1684, 1588, 1491, 1455, 1419, 1371, 1304, 1268, 1197, 1107, 1013, 960, 922, 834, 801, 745, 701, 597, 458, 429; ^1^H NMR (400 MHz, DMSO-d_6_) δ: 12.28 (s, 1H, –NH of indole), 7.04–7.91 (m, 8H, ArH), 2.96 (q, *J *= 7.6 Hz, 2H, –CH_2_), 1.89 (s, 3H, –CH_3_) 1.27 (t, *J *= 7.6 Hz, 3H, –CH_3_); ^13^C NMR (100 MHz, DMSO-d_6_) δ: 175.3, 169.2, 164.4, 154.0, 138.5, 136.2, 135.3, 132.0, 130.1, 128.8, 126.1, 125.2, 123.1, 121.6, 113.2, 23.9, 23.9, 13.1; APCI-MS m/z found for C_20_H_18_N_6_S: 374.46 (M^+^); Anal. calcd for C_20_H_18_N_6_S: C 64.15, H 4.85, N 22.44, S 8.56 found C 64.14, H 4.81 N 22.47, O 8.59.

(Z)-2,5-Dichloro-*N*-(1-(4-((Z)-(5-nitro-1*H*-indol-3-yl)diazenyl)phenyl)ethylidene)aniline (**DS-10**): Orange; Yield: 72% mp: 210–215 °C; R_f_ = 0.64 (ethyl acetate/methanol 7:3); IR (KBr, cm^−1^) ν_max_: 3347, 2923, 2854, 1704, 1619, 1517, 1468, 1328, 1252, 1167, 1113, 1062, 927, 895, 823, 785, 740, 687, 656, 590, 536, 449; ^1^H NMR (400 MHz, DMSO-d_6_) δ: 11.99 (s, 1H, –NH of indole), 9.97 (s, 1H, –CH(2) of indole), 8.43 (dd, *J*_1_ = 9.2 Hz, *J*_*2*_= 2.4 Hz, 1H, –CH(6) of indole), 8.37 (d, *J *= 2.4 Hz, 1H, –CH(7) of indole), 8.22 (d, *J *= 2.4 Hz, 1H, –CH(4) of indole), 7.98 (dd, *J*_1_ = 9.2 Hz, *J*_*2*_= 2.4 Hz, 1H, –CH=C–Cl), 7.58–7.63 (m, 2H, ArH), 7.18 (d, *J *= 8.4 Hz, 2H, ArH), 7.12 (d, *J *= 9.2 Hz, 1H, ArH), 6.81 (d, *J *= 2.4 Hz, 1H, ArH), 1.91 (s, 3H, CH_3_); ^13^C NMR (100 MHz, DMSO-d_6_) δ: 169.6, 158.7, 149.2, 140.2, 138.6, 138.0, 135.2, 132.7, 130.6, 129.7, 128.6, 128.8, 127.7, 126.6, 124.2, 123.2, 122.6, 121.6, 119.5, 115.3, 23.2; APCI-MS m/z found for C_22_H_15_Cl_2_N_5_O_2_: 452.3 (M^+^); Anal. calcd for C_22_H_15_Cl_2_N_5_O_2_: C 58.42, H 3.34 Cl 15.68, N 15.48, O 7.07 found C 58.45, H 3.37 N 15.51, O 7.06.

4-((Z)-(1-(4-((Z)-(1*H*-Indol-3-yl)diazenyl)phenyl)ethylidene)amino)-*N*-(pyridin-2-yl)benzenesulfonamide (**DS-11**): Maroon; Yield: 68% mp: 135–140 °C; R_f_ = 0.57 (ethyl acetate/methanol 7:3); IR (KBr, cm^−1^) ν_max_: 3221, 3055, 2924, 1677, 1623, 1593, 1530, 1495, 1459, 1386, 1331, 1245, 1130, 1083, 1007, 956, 830, 747, 676, 612, 564, 451; ^1^H NMR (400 MHz, DMSO-d_6_) δ: 11.84 (s, 1H, –NH of indole), 10.92 (s, 1H, –CH(2) of indole), 8.08 (d, *J *= 4.0 Hz, 2H, ArH), 7.60–7.65 (m, 3H, ArH), 7.48–7.52 (m, 3H, ArH), 7.05 (d, *J *= 8.4 Hz, 2H, ArH), 6.98–7.10 (m, 2H, –CH (5,6) of indole), 6.87–6.90 (m, 2H), 6.52–6.55 (m, 2H, ArH), 5.91–5.98 (m, 1H, –SO_2_NH), 1.89 (s, 3H, CH_3_); ^13^C NMR (100 MHz, DMSO-d_6_) δ: 169.8, 156.1, 155.2, 148.4, 137.6, 137.6, 136.5, 135.4, 133.2, 130.0, 128.3, 127.8, 123.6, 123.1, 123.1, 121.8, 120.9, 120.1, 117.8, 114.5, 112.5, 22.8; APCI-MS m/z found for C_27_H_22_N_6_O_2_S: 494.56 (M^+^); Anal. calcd for C_27_H_22_N_6_O_2_S: C 65.57, H 4.48, N 16.99, O 6.47 S 6.48 found C 65.59, H 4.49 N 16.96, O 6.51.

(Z)-*N*-(1-(4-((*Z*)-(1*H*-Indol-3-yl)diazenyl)phenyl)ethylidene)-4-chloro-2-nitroaniline (**DS-13**): Maroon; Yield: 65%; R_f_ = 0.49 (ethyl acetate/methanol 8:2); IR (KBr, cm^−1^) ν_max_: 3474, 3355, 3096, 3056, 2963, 2922, 2857, 1632, 1596, 1563, 1502, 1455, 1406, 1339, 1246, 1117, 1013, 959, 887, 817, 744, 645, 591, 521, 466, 416; ^1^H NMR (400 MHz, DMSO-d_6_) δ: 11.99 (s, 1H, –NH of indole), 9.97 (s, 1H, –CH(2) of indole), 8.43 (dd, *J*_1_ = 9.2 Hz, *J*_*2*_= 2.4 Hz, 1H, –CH–C–Cl), 8.37 (d, *J *= 2.0 Hz, 1H, –CH(4) of indole), 8.22 (d, *J *= 2.4 Hz, 1H, –CH(7) of indole), 7.98 (dd, *J*_1_ = 9.2 Hz, *J*_*2*_= 2.4 Hz, 1H, ArH), 7.58–7.63 (m, 2H, ArH), 7.18 (d, *J *= 8.4 Hz, 2H, ArH), 7.12 (d, *J *= 9.2 Hz, 1H, ArH), 6.81 (d, *J *= 2.4 Hz, 1H, –CH(5) of indole), 6.53 (dd, *J*_*1*_= 8.4 Hz, *J*_*2*_= 2.4 Hz, –CH(6) of indole), 1.91 (s, 3H, CH_3_); ^13^C NMR (100 MHz, DMSO-d_6_) δ: 169.3, 158.3, 149.3, 145.8, 140.7, 136.5, 135.3, 135.1, 134.2, 132.4, 131.2, 129.6, 127.5, 126.5, 125.1, 122.5, 121.7, 119.3, 115.4; APCI-MS m/z found for C_22_H_16_ClN_5_O_2_: 417.85 (M^+^); Anal. calcd for C_22_H_16_ClN_5_O_2_: C 63.24, H 3.86 Cl 8.48, N 16.76, O 7.66 found C 63.27, H 3.89 N 16.79, O 7.68.

4-((Z)-(1-(4-((Z)-(1*H*-Indol-3-yl)diazenyl)phenyl)ethylidene)amino)benzoic acid (**DS-14**): Maroon; Yield: 74%; mp: 205–210 °C; R_f_ = 0.71 (ethyl acetate/methanol 8:2); IR (KBr, cm^−1^) ν_max_: 3391, 2924, 1676, 1601, 1529, 1458, 1381, 1249, 1172, 1108, 1043, 1014, 962, 746, 604, 456, 416; ^1^H NMR (400 MHz, DMSO-d_6_) δ: 11.14 (s, 1H, –NH– of indole), 10.24 (s, 1H, –COOH), 9.12 (s, 1H, –CH(2) of indole), 7.87 (d, *J *= 8.8 Hz, 2H, ArH), 7.67 (d, *J *= 7.6 Hz, 1H, –CH(4) of indole), 7.37 (d, *J *= 8.0 Hz, 2H, ArH), 7.14–7.28 (m, 2H, ArH), 7.01–7.05 (m, 2H, ArH), 6.78–6.89 (m, 2H, –CH(5,6) of indole), 1.97 (s, 3H, CH_3_); ^13^C NMR (100 MHz, DMSO-d_6_) δ: 173.9, 169.5, 155.3, 152.4, 140.3, 138.2, 136.9, 135.4, 133.0, 129.2, 128.1, 126.7, 123.2, 122.3, 120.9, 119.4, 118.5, 115.2; APCI-MS m/z found for C_23_H_18_N_4_O_2_: 382.41 (M^+^); Anal. calcd for C_23_H_18_N_4_O_2_: C 72.24, H 4.74, N 14.65, O 8.37 found C 72.26, H 4.78 N 14.61, O 8.41.

*N*-((4-((Z)-(1-(4-((Z)-(5-Nitro-1*H*-indol-3-yl)diazenyl)phenyl)ethylidene)amino)phenyl) sulfonyl)acetamide (**DS-15**): Dark Maroon color; Yield: 64%; mp: 110–115 °C; R_f_ = 0.64 (ethyl acetate/methanol 8:2); IR (KBr, cm^−1^) ν_max_: 1685, 1620, 1509, 1512, 1458, 1419, 1373, 1319, 1242, 1188, 1130, 1067, 1033, 829, 740, 686, 624, 547; ^1^H NMR (400 MHz, DMSO-d_6_) δ: 11.96 (s, 1H, –NH– of indole), 9.93 (s, 1H, –CH(2) of indole), 8.56 (s, 1H, –NH– of sulfonamide), 8.41 (dd, *J*_1_ = 9.2 Hz, *J*_*2*_= 2.4 Hz, 1H, –CH(6) of indole), 8.35 (d, *J *= 2.8 Hz, 1H, –CH(4) of indole), 8.21 (d, *J *= 2.0 Hz, 1H, –CH(7) of indole), 7.96 (dd, *J*_1_ = 9.2 Hz, *J*_*2*_= 2.4 Hz, 2H, ArH), 7.49–7.59 (m, 2H, ArH), 7.10 (d, *J *= 9.2 Hz, 2H, ArH), 6.58–6.98 (m, 2H, ArH), 1.89 (s, 3H, CH_3_), 1.85 (s, 3H, COCH_3_); ^13^C NMR (100 MHz, DMSO-d_6_) δ: 173.9, 160.2, 158.3, 143.1, 140.2, 135.1, 130.3, 129.2, 126.6, 125.2, 123.6, 122.7, 120.4, 116.5, 23.2; APCI-MS m/z found for C_24_H_20_N_6_O_5_S: 504.51 (M^+^); Anal. calcd for C_24_H_20_N_6_O_5_S: C 57.14, H 4.00 Cl12.83, N 16.66, O 15.86, S 6.36 found C 57.17, H 4.03 N 16.68, O 15.81.

(Z)-4-Fluoro-*N*-(1-(4-((Z)-(5-nitro-1*H*-indol-3-yl)diazenyl)phenyl)ethylidene)aniline (**DS-16**): Dark Orange; Yield: 63%; mp: 150–152 °C; R_f_ = 0.61 (ethyl acetate/methanol 8:2); IR (KBr, cm^−1^) ν_max_: 3398, 3056, 2923, 1612, 1596, 1456, 1426, 1338, 1269, 1154, 1013, 962, 924, 883, 816, 743, 596, 449; ^1^H NMR (400 MHz, DMSO-d_6_) δ: 11.96 (s, 1H, –NH– of indole), 9.82 (s, 1H, –CH(2) of indole), 8.56 (s, 1H, indole), 8.41 (dd, *J*_*1*_= 9.2 Hz, *J*_*2*_= 2.4 Hz, 2H, ArH), 8.35 (d, *J *= 2.4 Hz, 1H), 8.20 (d, *J *= 2.4 Hz, 1H, indole), 8.11 (d, *J *= 7.6 Hz, 1H, ArH), 7.95 (dd, *J*_*1*_= 9.2 Hz, *J*_*2*_= 2.4 Hz, 2H, ArH), 7.53–7.59 (m, 2H, ArH), 7.11 (d, *J *= 9.2 Hz, 1H, ArH), 1.89 (s, 3H, CH_3_); ^13^C NMR (100 MHz, DMSO-d_6_) δ: 169.7 166.3, 163.2, 156.4, 148.2, 145.8, 142.2, 140.3, 137.5, 132.6, 129.4, 127.7, 126.0, 124.6, 123.6, 122.8, 121.6, 120.2, 119.2, 116.5, 115.4, 114.2, 23.7; APCI-MS m/z found for C_22_H_16_FN_5_O_2_: 401.39 (M^+^); Anal. calcd for C_22_H_16_FN_5_O_2_: C 65.83, H 4.02 F 4.73, N 17.45, O 7.97 found C 65.85, H 4.06 N 17.43, O 7.98.

(Z)-*N*-(1-(4-((Z)-(1*H*-Indol-3-yl)diazenyl)phenyl)ethylidene)pyrimidin-2-amine (**DS-17**): Maroon; Yield: 68%; mp: 275–280 °C; R_f_ = 0.57 (ethyl acetate/methanol 8:2); R_f_ = 0.54 (ethyl acetate/methanol 8:2); IR (KBr, cm^−1^) ν_max_: 3386, 1624, 1575, 1465, 1384, 1353, 1268, 1221, 1160, 801, 746, 653, 523, 421; ^1^H NMR (400 MHz, DMSO-d_6_) δ: 11.53 (s, 1H, –NH– of indole), 8.54 (s, 1H, –CH(2) of indole), 8.19 (d, *J *= 4.8 Hz, 2H, ArH), 6.82–7.95 (m, 7H, ArH), 6.51–6.53 (m, 2H, ArH), 1.89 (s, 3H, CH_3_); ^13^C NMR (100 MHz, DMSO-d_6_) δ: 171.9, 167.4, 160.2, 155.2, 143.2, 137.4, 136.7, 132.8, 130.6, 128.5, 126.4, 125.0, 123.2, 122.3, 120.3, 116.2, 23.1; APCI-MS m/z found for C_20_H_16_N_6_: 340.38 (M^+^); Anal. calcd for C_20_H_16_N_6_: C 70.57, H 4.74 N 24.69 found C 70.59, H 4.78 N 24.72.

4-((*Z*)-(1-(4-((Z)-(1H-Indol-3-yl)diazenyl)phenyl)ethylidene)amino)-*N*-(pyrimidin-2-yl) benzenesulfonamide (**DS-19**): Orange, Yield: 71%; R_f_ = 0.67 (ethyl acetate/methanol 8:2); IR (KBr, cm^−1^) ν_max_: 3422, 3358, 3106, 3038, 2935, 2870, 2811, 2736, 1706, 1585, 1494, 1440, 1410, 1384, 1328, 1260, 1152, 1091, 941, 834, 799, 741, 683, 569, 452, 416; ^1^H NMR (400 MHz, DMSO-d_6_) δ: 11.96 (s, 1H, –NH– of indole), 9.20 (s, 1H, –CH(2) of indole), 8.57 (s, 1H), 8.46 (d, *J *= 4.8 Hz, 2H, –CH of pyrimidine), 8.21 (d, *J *= 4.8 Hz, 2H, ArH), 7.94–7.97 (m, 2H, ArH), 7.58–7.61 (m, 3H, ArH), 6.98–7.1 (m, 3H, ArH), 6.54–6.56 (m, 3H, ArH), 5.98 (s, 1H, –NH), 1.89 (s, 3H, CH_3_); ^13^C NMR (100 MHz, DMSO-d_6_) δ: 169.7, 160.7, 158.3, 155.0, 153.2, 141.3, 139.6, 137.2, 135.6, 131.2, 128.3, 127.4, 126.2, 124.2, 122.2, 121.9, 121.1, 120.8, 116.1, 109.3, 23.1; APCI-MS m/z found for C_26_H_21_N_7_O_2_S: 495.55 (M^+^); Anal. calcd for C_26_H_21_N_7_O_2_S: C 63.02, H 4.27, N 19.79, O 6.44 S 6.47 found C 63.05, H 4.29, N 19.76, O 6.48.

(*Z*)-*N*1-(1-(4-((Z)-(1*H*-Indol-3-yl)diazenyl)phenyl)ethylidene)-*N2*-(naphthalen-1-yl)ethane-1,2-diamine (**DS-20**): Maroon; Yield: 72%; mp: 110–115 °C; R_f_ = 0.42 (ethyl acetate/methanol 8:2); IR (KBr, cm^−1^) ν_max_: 3387, 1675, 1582, 1528, 1481, 1407, 1277, 1118, 1018, 959, 753, 571, 438, 418; ^1^H NMR (400 MHz, DMSO-d_6_) δ: 11.51 (s, 1H, –NH– of indole), 8.71–8.88 (m, 1H, –CH(2) of indole), 8.24–8.37 (m, 2H, ArH), 8.04–8.10 (m, 1H, –CH of indole), 7.15–7.50 (m, 9H, ArH), 6.66 (d, *J *= 8.0 Hz, 1H, ArH), 6.53–6.58 (m, 1H, –NH–), 6.23–6.37 (m, 2H, ArH), 3.53–3.56 (m, 2H, CH_2_), 3.44–3.47 (m, 2H, CH_2_), 1.89 (s, 3H, CH_3_); ^13^C NMR (100 MHz, DMSO-d_6_) δ: 170.4, 159.8, 146.3, 140.0, 139.3, 135.7, 134.1, 132.0, 126.9, 125.6, 124.5, 123.5, 122.3, 122.0, 121.3, 121.1, 120.2, 118.2, 116.2, 114.3, 58.2, 47.5, 25.3; APCI-MS m/z found for C_28_H_25_N_5_: 431.21 (M^+^); Anal. calcd for C_28_H_25_N_5_: C 77.93, H 5.84 N 16.23, found C 77.95, H 5.81 N 16.27.

(*Z*)-*N*-(1-(4-((Z)-(5-Nitro-1*H*-indol-3-yl)diazenyl)phenyl)ethylidene)benzo[*d*]thiazol-2-amine (**DS-21**): Dark Orange; Yield: 69%; mp: 130–135 °C; R_f_ = 0.46 (ethyl acetate/methanol 8:2); IR (KBr, cm^−1^) ν_max_: 3567, 3340, 3119, 2922, 1668, 1618, 1523, 1455, 1388, 1355, 1162, 1121, 1061, 1017, 961, 899, 837, 785, 744, 655, 590, 480; ^1^H NMR (400 MHz, DMSO-d_6_) δ: 11.93 (s, 1H, –NH– of indole), 9.19 (s, 1H, CH=C–N–), 8.73 (d, *J *= 9.2 Hz, 1H, –CH(6) indole), 8.56 (s, 1H, –CH(4) indole), 8.41 (dd, *J*_1_ = 9.2 Hz, *J*_2_ = 2.4 Hz, 1H, –CH(7) indole), 8.16–8.20 (m, 2H, ArH), 7.41–7.97 (m, 2H, ArH), 7.31 (d, *J *= 8.0 Hz, 1H, –CH(4) benzothiazole), 7.19 (t, *J *= 7.2 Hz, 1H, –CH(5)– benzothiazole), 7.10 (d, *J *= 7.6 Hz, 1H, –CH(7)– benzothiazole), 6.96 (t, *J *= 7.6 Hz, 1H, –CH(6)– benzothiazole), 1.89 (s, 3H, CH_3_); ^13^C NMR (100 MHz, DMSO-d_6_) δ: 176.9, 155.9, 153.2, 148.5, 142.5, 140.6, 138.2, 136.8, 133.6, 130.9, 128.3, 126.9, 126.0, 123.8, 122.7, 121.9, 118.8, 118.7, 111.6, 23.2; APCI-MS m/z found for C_23_H_16_N_6_O_2_S: 440.47 (M^+^); Anal. calcd for C_23_H_16_N_6_O_2_S: C 62.72, H 3.66, N 19.08, O 7.26 S 7.28 found C 62.75, H 3.69 N 19.04, O 7.26.

### Antimicrobial evaluation

#### Determination of minimum inhibitory concentration (MIC)

The synthesized indole diazenyl Schiff bases were screened for antimicrobial activity through tube dilution method as per the reported procedure [41, 42]. The cefotaxime (antibacterial) and fluconazole (antifungal) were selected, as the standard drugs. The standard drugs and the test derivatives were dissolved in DMSO to make the stock solutions of the required concentration of 1000 μg/ml and further serially diluted in nutrient broth (for bacterial strains) and sabouraud dextrose broth (for fungal strains) to get the desired concentrations of (500, 250, 125, 62.5, 31.25, 15.62, 7.81, 3.90, 1.95 μg/ml). The each concentration of the test and standard compounds have been supplemented with 100 μl of microbial inoculum to give final inoculum size of 5 * 10^5^ colony forming units (CFU) ml^−1^ under sterile conditions. The all test tubes with different concentration of the test and standard compounds and microbial strains were incubated for the specified time (for bacterial cultures—24 h at 37 ± 2 °C; fungal cultures—7 days at 25 ± 2 °C).

#### Determination of minimum bactericidal/fungicidal concentration (MBC/MFC)

After MIC assessment, the indole derivatives (**DS1**–**DS21**), were additionally evaluated for MBC and MFC values. To the sterilized petri plates, added 100 µl of culture from each test tube which demonstrated no visible growth in MIC test tubes aseptically. The 10–15 ml of nutrient agar and Sabouraud dextrose agar was added to the petri plates for bacterial and fungal samples respectively with gentle shaking of plates in order to mix the culture throughout the media. Allowed the media to solidify. The petri plates were then incubated for the predefined time and temperature as referenced already for bacterial and fungal cultures respectively. The plates were then investigated visually for the development of microbial growth. The MBC and MFC were stated as the minimum concentration of the compounds in aliquots showing no visual growth after incubation.

### Cytotoxicity study

#### Cell culture

The cell lines used in study were initially procured from the National Centre for Cell Sciences (NCCS), Pune, India, and maintained in DMEM. The cell line was cultured in 25 cm^2^ tissue culture flask with DMEM supplemented with 10% FBS, sodium bicarbonate, l-glutamine, and antibiotic solution containing: streptomycin (100 μg/ml), penicillin (100 U/ml). Cultured cell line was kept at 37 °C in a humidified 5% CO_2_ incubator (VWR, USA).

#### MTT cell proliferation assay

The compounds found to have good antimicrobial potential were then screened for their cytotoxicity using MTT (3,4,5-dimethylthiazol-2-yl)-2-5-diphenyltetrazolium bromide) assay [43, 44]. 1 × 10^4^ cells/well were seeded in 100 µl DMEM/MEM, supplemented with 10% FBS in each well of 96-well microculture plates and incubated for 24 h at 37 °C in a CO_2_ incubator. After incubation, all the prepared/synthesized compounds were added to the cells at 10, 25, 50 and 100 µg concentrations for 48 h. After 48 h of drug treatment, 10 µl MTT (5 mg/ml) was added to each well and the plates were further incubated for 4 h. Then the supernatant from each well was carefully removed, formazan crystals were dissolved in 100 µl of DMSO and absorbance at 570 nm wavelength was recorded on an ELISA reader. The IC_50_ value was calculated using the linear regression equation i.e. Y = Mx + C. Here, Y = 50, M and C values were derived from the viability graph. The assay was performed in triplicate.

#### Molecular docking

The novel indole diazenyl Schiff bases were subjected to dock in the active site of DNA gyrase enzyme using Schrodinger Glide software. The 3D-crystal structure of the ATP binding site of *E. coli* GyrB in complex with pyrimido [4,5-*b*]indole derivative (PDB ID: 4KFG, resolution 1.6 Å) had been used for the modelling studies and was retrieved from Protein Data Bank (http://www.rcsb.org/pdb/home/home). The target derivatives were investigated for the theoretical binding mode at the ATP binding site to understand the ligand-receptor possible intermolecular interactions in detail using molecular docking modelling. The selected protein structure was prepared using the Protein Preparation Wizard executed in Schrödinger Suite 2018-1. Crystallographic water molecules with fewer than three hydrogen bonds were deleted. Hydrogen atoms were added to the protein structure corresponding to a pH value of 7. The restrained minimization was performed until the heavy atoms RMSD reached a maximum cut-off to 0.30 Å. The active site was defined with a 20 Å radius around the ligand present in the crystal structure and a grid box was generated at the centroid of the active site. Low-energy conformations of all ligands were docked into the catalytic pocket of the 4KFG protein in extra precision mode (Glide, Schrödinger 2018-1) without applying any constraints. The best docked structures were selected based on the Glide score function, Glide energy and Glide energy model [45, 46].

#### ADME properties

ADME properties were calculated using Qikprop v3.5 tool of Schrödinger. It predicts both physicochemically significant descriptors and pharmacokinetic relevant properties. QikProp provides ranges for comparing a particular molecule’s properties with those of 95% of known drugs. Qikprop evaluates the acceptability of analogs based on Lipinski’s rule of five, which is essential to ensure drug-like pharmacokinetic profile while using rational drug design. All the analogs were neutralized before being used by Qikprop.

## Conclusion

In quest of effective antimicrobial and cytotoxic agents, a series of indole hybridized diazenyl derivatives (**DS-1**–**DS-21**) was efficiently prepared and characterized. The synthesized derivatives were evaluated for antimicrobial activities against various pathogenic bacterial and fungal strains. Most of the synthesized derivatives especially **DS-6**, **DS-10**, **DS-14**, **DS-20** and **DS-21** demonstrated excellent antibacterial activity against Gram-negative bacteria particularly *E. coli* and *K. pneumonia*. The derivatives **DS-14** and **DS-20** have shown good cytotoxicity against breast cancer cell line and moderate activity against human colorectal carcinoma cell line. Most of the tested derivatives were found to be non-toxic to the leukemic cancer cell line. The synthesized derivatives revealed high safety level by exhibiting very low cytotoxicity against the normal cell line. The molecular docking studies validated the outcome results from the antimicrobial activity and signifies the potential of these derivatives as DNA gyrase enzyme inhibitors. So, these compounds can be modified further for the development of new anticancer and antimicrobial agents.

## Data Availability

Provided in manuscript.

## Abbreviations

DMEM: Dulbecco’s Modified Eagle Medium
MEM: minimum essential medium
FBS: fetal bovine serum
MTT: 3-(4,5-dimethylthiazol-2-yl)-2,5-diphenyltetrazolium bromide
MIC: minimum inhibitory concentration
MBC: minimum bactericidal concentration
MFC: minimum fungicidal concentration
ADME: absorption, distribution, metabolism, excretion
ELISA: enzyme-linked immunosorbent assay
HEK-293: human embryonic kidney cells
